# Prognosis and role of clinical and imaging features in patients with malignant pericardial effusion: a single-center study in China

**DOI:** 10.1186/s12872-021-02331-9

**Published:** 2021-11-26

**Authors:** Shucai Wang, Jiazheng Zhao, Chanchan Wang, Ning Zhang

**Affiliations:** 1 Department of Cardiology, The Fourth Hospital of Hebei Medical University, 12 Health Road, Shijiazhuang, Hebei 050011 People’s Republic of China; 2 Department of Orthopedics, The Fourth Hospital of Hebei Medical University, 12 Health Road, Shijiazhuang, Hebei 050011 People’s Republic of China

**Keywords:** Malignant pericardial effusion, Clinical features, Imaging performance, Treatment, Prognosis

## Abstract

**Background:**

The diagnosis of malignant pericardial effusion (MPE) is often associated with a poor prognosis, but due to the complexity and unspecific nature of MPE patients' clinical manifestations, imaging often performs an essential role in diagnosis and prognosis.

**Methods:**

Patients diagnosed with MPE between 2013 and 2018 at one tumor hospital were included and followed up. The data covered the basic clinical features, imaging findings, treatments and prognosis of patients with MPE, and the factors that may have affected the prognosis were explored.

**Results:**

A total of 216 patients with MPE were included with the median age of 60 years. The most common primary cancer type was lung cancer (73.6%), the most common symptom was dyspnea (62.9%) and the most common abnormal electrocardiogram finding was sinus tachycardia (42.1%). The median survival time of the 216 patients with MPE was 13.7 months. The factors affecting prognosis were echocardiographic fluid signs (*HR* = 2.37, *P* = 0.010), electrocardiographic evidence of sinus tachycardia (*HR* = 1.76, *P* = 0.006) and echocardiographic evidence of cardiac tamponade (*HR* = 3.33, *P* < 0.001).

**Conclusions:**

MPE has complex clinical manifestations and an unsatisfactory prognosis. Echocardiographic fluid signs, electrocardiographic evidence of sinus tachycardia, and echocardiographic evidence of cardiac tamponade are independent risk factors affecting prognosis.

## Background

Under physiological conditions, the pericardial cavity contains a small amount of liquid (approximately 50 ml). Pericardial effusion is excess liquid in the pericardial cavity [1]. Malignant tumor is a common cause of pericardial effusion, either during invasion or treatment of the tumor [2]. Pericardial effusion, as a serious complication in patients with advanced cancer, is significantly associated with reduced survival [3]. The diagnosis of malignant pericardial effusion (MPE) is often associated with a poor prognosis, but due to the complexity and unspecific nature of MPE patients' clinical manifestations, imaging often performs an essential role in diagnosis and prognosis [4].

Accordingly, in this study, we analyzed the clinical data of 216 patients diagnosed with MPE and explored the prognostic factors affecting MPE to provide a reference and guidance for the clinical diagnosis and management of MPE.

## Methods

### Study population

Patients diagnosed with MPE between 2013 and 2016 from one tumor hospital in China were included in this study. This study was approved by the Ethics Committee of the Fourth Affiliated Hospital of Hebei Medical University. The selection criteria were: (1) patient had a history of malignancy; (2) patient was diagnosed with MPE by pericardial cytopathology; (3) Non-oncological causes of pericardial effusion such as radiation pericarditis and chemotherapy drug-induced cardiotoxicity were excluded [5, 6].

Echocardiographic parameters were used for diagnosing cardiac tamponade: RA-compression, RV-compression, swinging heart, exaggerated respiratory variability (> 25%) in mitral inflow velocity, inspiratory decrease and expiratory increase in pulmonary vein diastolic forward flow or combination.

### Data collection

In this study, the case data we collected included the following aspects: (1) basic information of the patients: age, gender, primary cancer, comorbidities (liver cirrhosis, diabetes and hypertension); (2) diagnostic information: time of diagnosis, progression-free survival at the first diagnosis, time of death, etc.; (3) clinical manifestations: signs, symptoms; (4) auxiliary examinations: important results of electrocardiogram, X-ray, echocardiography, chest CT; (5) treatment: systemic chemotherapy, pericardial chemotherapy, only symptomatic treatment; and (6) efficacy: whether remission after treatment, recurrence after remission, survival at the end of the follow-up period. If the patients died, the specific time of death was recorded.

### Follow-up

Survival information was obtained from patients or relatives by telephone follow-up. The deadline for follow-up was December 31, 2020, and the follow-up rate was 100%. Overall survival (OS) was defined as the time from diagnosis to the time of any cause of death or the deadline for follow-up.

### Statistical analysis

The sex, age, primary cancer, signs, imaging findings, and treatment status of the 216 MPE patients were analyzed. The data were analyzed using SPSS 25.0 software (SPSS Inc., Chicago, IL, USA). Continuous variables that conformed to a normal distribution were expressed as the mean (standard deviations [SD]) and those that failed to conform were expressed as the median (inter quartile range [IQR]). Categorical variables were expressed as proportions (%) and frequencies (n). The prognostic analyses were performed using the univariate Cox analysis and multivariate Cox analysis. All statistical tests were two-sided and *P* < 0.05 was considered significant.

## Results

### Population characteristics

Table 1 details the population characteristics of the study participants. Of the 216 patients, 123 were male and 93 were female. The median age was 60 years old, and there was no significant difference in age between men and women (*P* = 0.191).

**Table 1:** Basic clinical features of patients with malignant pericardial effusion

Characteristics	Number of cases	Results
Age (years) (median, IQR)	216	60 (10)
Male (col %)	123	56.9
*Signs (mean, SD)*	216	
Body temperature (°C)		37.1 ± 1.3
Heart rate (times/min)		108 ± 12
Systolic pressure (mmHg)		116 ± 14
Diastolic blood pressure (mmHg)		75 ± 15
*Symptoms (col %)*	175	
Pericardial fluid sign		131 (74.9)
Shortness of breath/difficulty breathing		110 (62.9)
Chest tightness		72 (41.1)
Chest pain		38 (21.7)
Powerless		9 (5.1)
Pulsus paradoxus		18 (10.3)
Wound when visiting other diseases		18 (10.3)

The highest proportion of primary cancer type was lung cancer, which accounted for a total of 159 patients (73.6%), followed by breast cancer in 30 patients (13.9%) and esophageal cancer in 17 patients (7.9%). Among the 159 lung cancer cases, 119 were adenocarcinoma, 19 were squamous carcinoma, 11 were small cell carcinoma and 10 were other histological types.

### Basic clinical features

Table 1 also shows the basic clinical features of the study participants. Among the 216 patients, 175 were symptomatic. The major clinical manifestations of MPE patients were pericardial fluid sign (74.9%) and shortness of breath/dyspnea (62.9%). In addition, 10.3% (18/175) of patients were found to have MPE because of other diseases.

### Auxiliary examination performance

Table 2 shows the auxiliary examination performance of the participants. The electrocardiographic data of 216 patients in this study showed that the most common ECG change was sinus tachycardia (42.1%). The positive rates of X-ray and ultrasonographic diagnosis of pericardial effusion in the 216 patients were 76.4% (165/216) and 88.9% (192/216), respectively. There were 136 MPE patients with CT data in this study. The positive rate of diagnosis of pericardial effusion was 79.4% (85/136), and 91.2% (124/136) of patients showed mediastinal lymphadenopathy. 80 of these samples were positive for both pericardial effusion and mediastinal lymphadenopathy presentations.

**Table 2:** Auxiliary examination performance of patients with malignant pericardial effusion

Auxiliary examination performance	Number of cases	Result
*Electrocardiogram (col %)*	216	
Sinus tachycardia		91 (42.1)
ST-T anomaly		61 (28.2)
Low QRS voltage		38 (17.6)
Electric alternation		46 (21.3)
Atrial fibrillation		16 (7.4)
*X-Ray (col %)*	216	
Pericardial effusion		165 (76.4)
Increased heart shadow		173 (80.1)
Pleural effusion		112 (51.9)
*Echocardiography (col %)*	216	
Pericardial effusion		192 (88.9)
Pleural effusion		155 (71.8)
Pericardial thickening		57 (26.4)
*Chest CT (col %)*	136	
Pericardial effusion		108 (79.4)
Pleural effusion		85 (62.5)
Pericardial thickening		55 (40.4)
Mediastinal lymphadenopathy		124 (91.2)

### Treatment and outcome

Of the 216 patients, 139 (64.4%) received systemic chemotherapy, 119 (55.1%) received intracardiac chemotherapy, and 52 (24.1%) received symptomatic treatment. The in-hospital mortality rate was 9.3% (20/216). The 1-, 3-, and 5-year survival rates after discharge were 80.1% (173/216), 61.6% (133/216) and 53.2% (115/216), respectively.

### Univariate Cox analysis of related prognosis

**Fig. 1:**
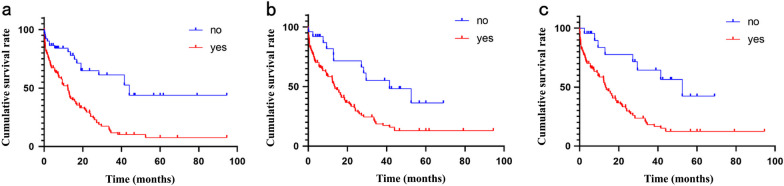
Kaplan–Meier curves of patients with MPE. **a** Cardiac tamponade, **b** echocardiographic fluid signs, **c** sinus tachycardia

The patient's median OS was 13.7 months. As shown in Table 3 and Fig. 1, univariate Cox analysis showed that echocardiographic fluid signs (*P* = 0.001), electrocardiographic evidence of sinus tachycardia (*P* = 0.002) and echocardiographic evidence of cardiac tamponade (*P* < 0.001*)* had a significant effect on patient survival.

**Table 3:** Univariate Cox analysis of prognosis in patients with malignant pericardial effusion

Characteristics	Results	
	HR (95%CI)	*P* values
Age (years)	1.032(0.885–1.201)	0.886
Gender (male)	0.844(0.792–1.101)	0.292
Lung cancer	1.547(0.621–2.145)	0.638
Comorbidities	1.258(0.926–1.685)	0.245
Treatment	0.942(0.496–1.635)	0.784
Sinus tachycardia	1.720(1.145–2.425)	0.002
Increased heart shadow	1.368(0.915–1.783)	0.216
Echocardiographic fluid signs	2.465(1.358–4.820)	0.001
Mediastinal lymphadenopathy	0.764(0.586–1.169)	0.373
Cardiac tamponade	3.625(2.365–5.721)	< 0.001

### Multivariate Cox analysis of related prognosis

We included the factor of *P* < 0.05 in the single-factor analysis of survival into the Cox regression model for multivariate analysis. As shown in Table 4, there was still a significant correlation among echocardiographic fluid signs (*HR* = 2.37, *P* = 0.010), electrocardiographic evidence of sinus tachycardia (*HR* = 1.76, *P* = 0.006) and echocardiographic evidence of cardiac tamponade (*HR* = 3.33, *P* < 0.001), and they were independent factors influencing patient survival.

**Table 4:** Multivariate Cox analysis of prognosis in patients with malignant pericardial effusion

Characteristics	Results	
	HR (95%CI)	*P* values
Echocardiographic fluid signs	2.372 (1.228–4.580)	0.010
Sinus tachycardia	1.755 (1.179–2.613)	0.006
Cardiac tamponade	3.328 (2.038–5.436)	< 0.001

## Discussion

MPE is a serious complication of advanced malignant tumor patients and indicates a poor prognosis [7]. This report describes a study with a large number of patients with MPE. The primary cancers that cause MPE were mainly lung cancer, breast cancer and esophageal cancer, which were similar to reports from other studies [8]. This systematic summary and analysis of the basic clinical features, imaging findings, treatments and prognoses of MPE will help to enhance physicians’ comprehensive understanding of the disease.

The normal pericardial cavity contains up to 50 ml of liquid, and cancer cells can invade the pericardial cavity by direct invasion or by blood or lymphatic metastasis, resulting in a large amount of malignant liquid accumulation. The clinical manifestations of MPE patients are related to the rate of fluid accumulation. When pericardial fluid accumulates rapidly, only 200 ml of liquid will cause significant hemodynamic changes [9]. When pericardial fluid slowly increases, the pericardium slowly relaxes without significant changes in pericardial cavity pressure. Even if the pericardial cavity contains up to 2000 ml of fluid, the patient may have no obvious clinical symptoms [10]. Therefore, the clinical manifestations of MPE patients lack specificity. This study showed that the most common symptom of MPE was dyspnea (62.9%), which may be related to pericardial fluid accumulation and impaired ventricular filling. According to the physical examination results, 74.9% (131/175) of patients had signs of pericardial fluid, but only 21.7% (38/175) had a specific clinical manifestation of chest pain, further confirming the complexity and diversity of MPE clinical manifestations.

Abnormal ECG changes in patients with MPE are usually associated with myocardial injury and pericardial effusion under the pericardium. Common abnormal changes are ST-T segment abnormalities, sinus tachycardia, low QRS voltage and electrical alternation [11]. Abnormalities in the ST-T segment can reflect the extent of myocardial damage. This study showed that 28.2% (61/216) of patients had ST-T segment abnormalities, which we presumed to be related to direct tumor invasion and pericardial hydraulic compression. Regardless of the cause of effusion, effusion itself causes a conduction short circuit, resulting in a low voltage. This study showed that only 17.6% (38/216) of patients had low QRS voltage. Studies have shown that a low QRS voltage is not directly related to effusion itself and is associated with a decrease in left ventricular stroke power caused by fluid accumulation [12].

Sinus tachycardia is the most common compensatory mechanism in patients with pericardial effusion. As the amount of fluid increases, diastolic function is limited, ventricular filling is reduced, ejection fraction decreases, and the compensatory heart rate increases. These are also the most common abnormal electrocardiogram findings in MPE patients. Our study showed that 42.1% (91/216) of patients developed sinus tachycardia, which is slightly lower than in previous reports [13]. This may be related to the reduction in sinus node function caused by treatments such as radiotherapy and chemotherapy. Alternate or arbitrary combinations of P, QRS, ST, or T waveforms are specific ECG findings in a large number of patients with pericardial effusion. This study showed that 21.3% (46/216) of patients had evidence of electrical alternans. And other studies have shown that electrocardiographic evidence of electrical alternation has lower sensitivity in the diagnosis of pericardial effusion [14].

Echocardiography is the sensitive imaging technique for detecting pericardial effusion, and it perfectly shows a large number of pericardial effusion features [15]. And chest CT also has an irreplaceable advantage in the diagnosis of pericardial cavity occupation [16]. In our study, although chest CT had a diagnostic rate of only 79.4% for MPE yet it didn't indicate that the diagnostic efficacy of CT was weaker than that of echocardiography. It was difficult to ensure that all imaging examinations were performed within the same disease stage for each patient, so the corresponding positive diagnostic rates were not highly comparable. We speculated that the low diagnostic rate might be due to the fact that patients with tumors underwent routine chest CT at an early stage without demonstrating the pericardial effusion at that time. Diagnostic efficacy aside, compared to chest CT, echocardiography provides additional information on the impairment of cardiac function due to pericardial effusion [17].

This study confirmed the unsatisfactory prognosis of MPE. The in-hospital mortality rate was 9.3% (20/216), and the 5-year mortality rate after discharge was 46.8% (101/216). The Cox regression results showed that echocardiographic fluid signs (*HR* = 2.37, *P* = 0.010), electrocardiographic evidence of sinus tachycardia (*HR* = 1.76, *P* = 0.006) and echocardiographic evidence of cardiac tamponade (*HR* = 3.33, *P* < 0.001) had a significant relationship with patient survival. Moreover, they were all independent factors influencing the survival of patients. The treatment methods recorded in this study are more directed to the treatment of primary disease. It has been reported that systemic chemotherapy may be effective for lymphoma and breast cancer, and pericardial chemotherapy is the preferred treatment for lung cancer [18].

Lung cancer, a malignancy with high morbidity and mortality, was also the highest proportion of primary cancer type in this study. In recent years, the role of immune checkpoint inhibitors in the treatment of pericardial effusion caused by lung cancer is gradually being revealed. Cai et al. observed that immune checkpoint inhibitor, represented by nivolumab, reduced pericardial effusion in patients with advanced non-small cell lung cancer after 11 cycles of treatment [19].

As a single-center investigation, this study has limitations and the conclusions require confirmation in a large sample and multi-center trial. In addition, the results of imaging examinations that are not included in the same disease stage may restrict the comparison of diagnostic efficacy.

## Conclusion

In summary, MPE has complex clinical manifestations, an unsatisfactory prognosis, and a lack of effective treatment. Due to the relatively low incidence of the disease, more clinical studies are needed to determine the risk factors for and to predict the incidence of the disease in order to achieve early diagnosis, individualization and comprehensive treatment for MPE.

## Data Availability

The datasets used and/or analysed during the current study available from the corresponding author on reasonable request.
